# Sensitivity of the Wondfo One Step COVID-19 test using serum samples

**DOI:** 10.6061/clinics/2020/e2013

**Published:** 2020-05-25

**Authors:** Vera Aparecida dos Santos, Mayra Matias Rafael, Ester Cerdeira Sabino, Alberto José da Silva Duarte

**Affiliations:** I Divisao do Laboratorio Central (DLC), LIM 03, Hospital das Clinicas HCFMUSP, Faculdade de Medicina, Universidade de Sao Paulo, Sao Paulo, SP, BR; II Departamento de Molestias Infecciosas e Parasitarias, Faculdade de Medicina FMUSP, Universidade de Sao Paulo, São Paulo, SP, BR; III Departamento de Patologia, Faculdade de Medicina FMUSP, Universidade de Sao Paulo, Sao Paulo, SP, BR

To the editor,

There have been more than 40,000 reported cases of coronavirus disease (COVID-19) in Brazil until May 8, 2020. Recurrent testing among health care professionals is the key step to be undertaken by hospitals, as completely asymptomatic infection might occur among these professionals. Hospital das Clinicas is the main hospital dedicated to COVID-19 response in the city of Sao Paulo, with 224 intensive care unit beds.

We have set up a PCR testing program to detect COVID-19 among symptomatic health care professionals in the early phase of the epidemic. However, an antibody-based screening program was difficult to establish because of the lack of available antibody tests in Brazil. Recently, we received a donation of Wondfo One Step COVID-19 rapid test kits (Guangzhou, China) from a Brazilian company. The test is generally performed on capillary blood samples, but it can also be performed on peripheral blood, plasma, and serum.

We collected 47 paired samples (capillary and serum) from health care workers within 15 days of their confirmed diagnosis of COVID-19 by PCR to validate the test. The results were compared with those of the Euroimmun IgG EIA test (Luebeck, Germany).

The results of the test have been summarized in Table 1. The rapid test showed a low sensitivity when performed with capillary blood (55%); however, when tested on serum samples it exhibited a similar sensitivity to that of the Euroimmun IgG EIA assay (96%).

**Table 1:** Comparison of the Wondfo One Step COVID‐19 test (capillary and serum samples) and Euroimmun IgG EIA test results.

Wondfo One Step COVID-19 test	Euroimmun IgG test	
Capillary blood	Positive	Negative
Positive	26	
Negative	19	2
Serum		
Positive	45	
Negative		2

Recently, the government of Brazil has purchased a considerable number of Wondfo rapid test kits. Our results indicate that this test should not be performed using capillary blood but rather be performed with plasma or serum.

